# Rapid antigen diversification through mitotic recombination in the human malaria parasite *Plasmodium falciparum*

**DOI:** 10.1371/journal.pbio.3000271

**Published:** 2019-05-13

**Authors:** Xu Zhang, Noah Alexander, Irina Leonardi, Christopher Mason, Laura A. Kirkman, Kirk W. Deitsch

**Affiliations:** 1 Department of Microbiology and Immunology, Weill Cornell Medical College, New York, New York, United States of America; 2 Department of Physiology and Biophysics, Weill Cornell Medical College, New York, New York, United States of America; 3 Jill Roberts Center for Inflammatory Bowel Disease, Weill Cornell Medical College, New York, New York, United States of America; 4 HRH Prince Alwaleed Bin Talal Bin Abdulaziz Alsaud Institute for Computational Biomedicine, Weill Cornell Medical College, New York, New York, United States of America; 5 Feil Family Brain and Mind Research Institute, Weill Cornell Medical College, New York, New York, United States of America; 6 WorldQuant Initiative for Quantitative Prediction, Weill Cornell Medical College, New York, New York, United States of America; 7 Department of Internal Medicine, Division of Infectious Diseases, Weill Cornell Medical College, New York, New York, United States of America; University of Melbourne, AUSTRALIA

## Abstract

Malaria parasites possess the remarkable ability to maintain chronic infections that fail to elicit a protective immune response, characteristics that have stymied vaccine development and cause people living in endemic regions to remain at risk of malaria despite previous exposure to the disease. These traits stem from the tremendous antigenic diversity displayed by parasites circulating in the field. For *Plasmodium falciparum*, the most virulent of the human malaria parasites, this diversity is exemplified by the variant gene family called *var*, which encodes the major surface antigen displayed on infected red blood cells (RBCs). This gene family exhibits virtually limitless diversity when *var* gene repertoires from different parasite isolates are compared. Previous studies indicated that this remarkable genome plasticity results from extensive ectopic recombination between *var* genes during mitotic replication; however, the molecular mechanisms that direct this process to antigen-encoding loci while the rest of the genome remains relatively stable were not determined. Using targeted DNA double-strand breaks (DSBs) and long-read whole-genome sequencing, we show that a single break within an antigen-encoding region of the genome can result in a cascade of recombination events leading to the generation of multiple chimeric *var* genes, a process that can greatly accelerate the generation of diversity within this family. We also found that recombinations did not occur randomly, but rather high-probability, specific recombination products were observed repeatedly. These results provide a molecular basis for previously described structured rearrangements that drive diversification of this highly polymorphic gene family.

## Abstract 2

Malaria parasites have a remarkable capacity to continuously generate genetically diverse surface antigens, helping them to evade the host immune response. This study reveals the unusual recombination mechanism which accelerates the diversification of this gene family in Plasmodium falciparum, the most severe of the human malarial parasites.

## Introduction

Despite recent progress, malaria remains an infectious disease that inflicts a tremendous health and economic burden on many regions of the developing world, in particular sub-Saharan Africa [1]. The lack of a truly efficacious vaccine and the ever-present threat of drug resistance represent continuing challenges for public health organizations. Malaria is caused by eukaryotic parasites of the genus *Plasmodium*, with *P*. *falciparum* being the most virulent of the human infectious species. One unique aspect of *P*. *falciparum* biology is the tremendous genome plasticity displayed by parasites that are actively circulating within regions of the world endemic for the disease [2]. Much of this diversity is found within the subtelomeric regions of the 14 chromosomes where genes that encode variant surface antigens reside [3]. These chromosomal regions can extend for over 100 kb from the chromosome end and display a unique structure that includes telomere-associated repeat elements (TAREs) and members of several hypervariable gene families, including *var*, *rifin* (repetitive interspersed family), *stevor* (subtelomeric variant open reading frame), and *Pfmc-2TM* (*P*. *falciparum* Maurer’s cleft-2 transmembrane domain protein). Each of these families contain multiple members that display great sequence diversity, both within a single parasite’s genome and also when comparing the genomes of different geographical isolates [2,4–6]. The best characterized of these gene families is *var*, with the total number of *var* genes within the genome of any given parasite varying between approximately 50 and 90 [3].

*var* genes encode the primary virulence determinant displayed on the surface of infected red blood cells (RBCs), a protein referred to as *P*. *falciparum* erythrocyte protein 1 (PfEMP1) [7–9]. PfEMP1 is a single-transmembrane-domain–containing protein that crosses the RBC membrane, extending into the extracellular space, where it interacts with molecules found on the endothelial surface of postcapillary venules. The structure of PfEMP1 includes a series of adhesive domains within the extracellular portion of the protein enabling it to anchor the infected cell to the endothelium, thereby removing it from systemic circulation and preventing its movement through the spleen, leading to many of the severe complications of malaria, including cerebral malaria and pregnancy-associated malaria [10]. In both cases, adhesion within specific organs results from the display on the RBC surface of a form of PfEMP1 that binds to endothelial receptors found in these specific tissues.

The placement of PfEMP1 on the infected RBC surface makes it susceptible to the humoral immune response of the infected individual, and people carrying *P*. *falciparum* parasites readily develop anti-PfEMP1 antibodies [11,12]. This results in rapid clearance of parasitized cells and a substantial reduction in parasitemia. However, parasites are capable of switching which *var* gene is expressed, thereby displaying a different form of PfEMP1 that is not recognized by the preceding antibody response. This enables the generation of a new wave of parasitemia and promotes chronic infections that can last over a year. This process is referred to as antigenic variation and underlies many of the difficulties in creating effective vaccines against malaria [13].

Antigenic variation is entirely dependent on extensive sequence diversity within the genes encoding antigenic proteins, both within the genome of an individual parasite, which enables the maintenance of a chronic infection, and also between the gene repertoires of different parasite lines, thereby enabling multiple, sequential infections of a single host. This extensive diversity between parasite isolates explains why long-term immunity against malaria requires multiple infections over several years and provides protection against severe disease rather than preventing new infection [14,15]. Comparison of multiple complete genome assemblies from different geographical isolates indicates that the subtelomeric regions containing the multicopy, variant-antigen–encoding genes undergo much more rapid diversification than the rest of the “core” genome, in which single-copy housekeeping genes reside [3]. Two elegant studies employing the generation of extensive “clone trees” of laboratory-reared parasite lines showed that this process includes frequent and extensive ectopic recombination within chromosomal regions containing variant-antigen–encoding genes during asexual, mitotic replication [16,17]. This results in the creation of chimeric genes that encode mosaic sequences but that maintain open reading frames and basic protein domain structures. These observations provided key insights into how parasites continuously generate extensive sequence diversity and avoid the immune response of their human hosts, including a prominent role for homologous recombination (HR) between *var* genes. However, how this process is regulated and why subtelomeric regions are hyper-recombinogenic was not understood.

To examine the mechanisms of DNA repair in subtelomeric regions and *var* gene recombination more closely, we investigated the parasite’s response to targeted DNA double-strand breaks (DSBs) within a subtelomeric region of chromosome 12. When provided with a highly homologous template for repair by HR, these breaks were efficiently repaired without major alterations to the structure of the genome. However, when a suitable sequence was not available to serve as template for repair by HR, parasites frequently employed de novo telomere addition or “telomere healing” to stabilize the chromosome end and thus maintain genome integrity. While this process efficiently prevents further degradation of the chromosome and protects the core genome, it also generates a “free” DNA fragment consisting of the subtelomeric domain between the site of the break and the original chromosome end. We observed that this free DNA fragment is highly recombinogenic, resulting in a cascade of ectopic HR events that generated chimeric *var* genes on multiple chromosomes. Thus, a single DNA break within a subtelomeric region can lead to the creation of multiple new *var* genes, providing a driving force for diversification of this gene family. The role of telomere healing in creating the hyper-recombinogenic, free DNA fragment also provides a molecular basis for why these regions of the genome display much more rapid diversification than the more central regions of the chromosomes. This type of combinatorial recombination at chromosome ends has parallels in other eukaryotic organisms, including cancer cells, suggesting it is based on evolutionarily conserved mechanisms of DNA repair found throughout the eukaryotic lineage. The discovery of its role in accelerating *var* gene recombination indicates that it might represent a common mechanism for antigen diversification in a number of pathogens that have a similar arrangement of antigen-encoding genes near their chromosome ends.

## Results

### Repair of DNA DSBs by HR within a subtelomeric region of chromosome 12

**Fig 1:**
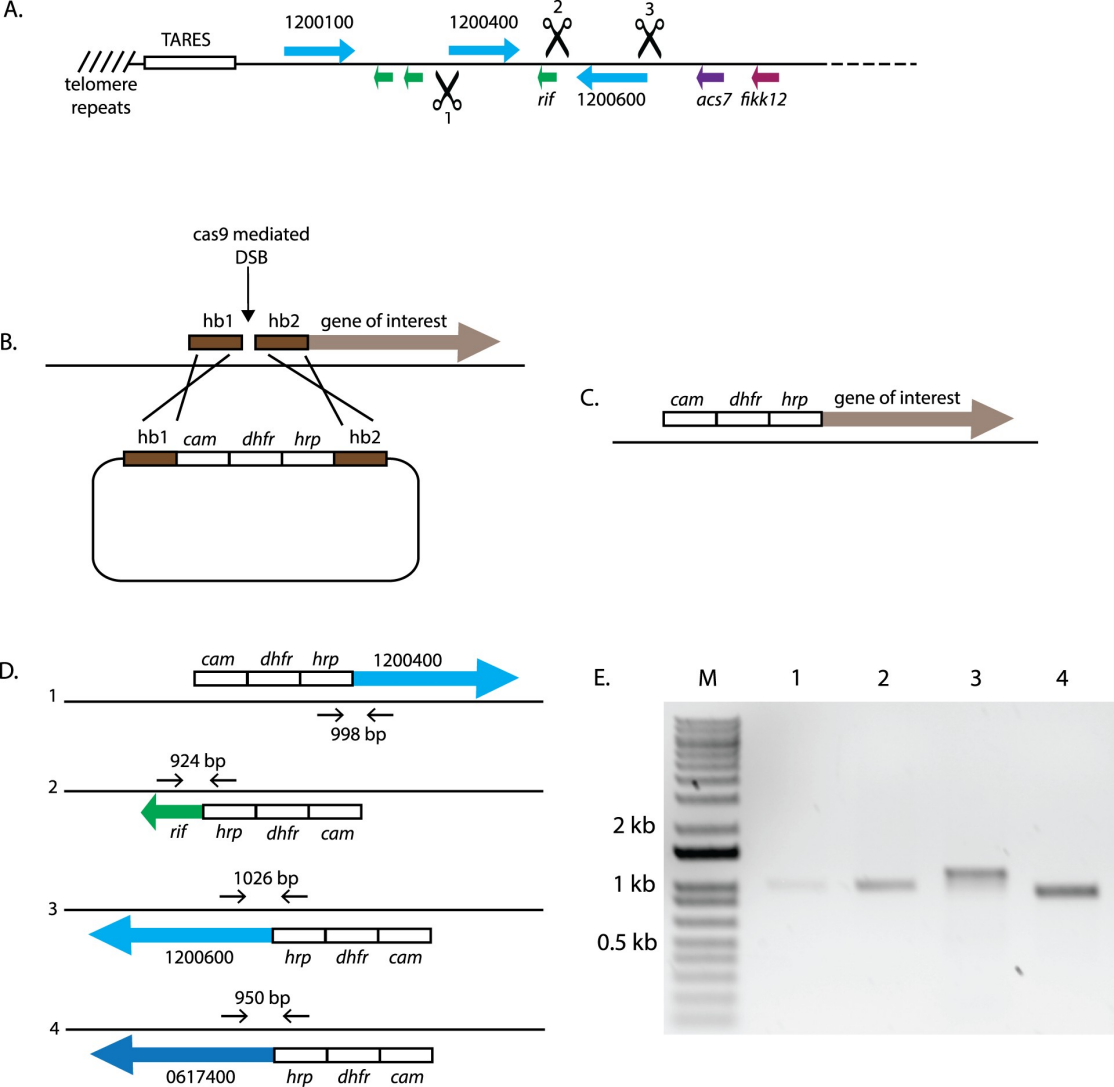
Repair of DSBs within a subtelomeric region of chromosome 12 by HR. (A) A map of the gene structure of the “left” end of chromosome 12. The annotation numbers for each *var* gene are shown, and the positions of the genes encoding *acs7* and a member of the FIKK family (*fikk12*) are labelled. Scissors represent sites for cleavage by Cas9. Hashed lines represent telomeric repeats and the position of TAREs are shown. (B) A schematic representation of a plasmid containing hb1 and hb2 with significant sequence identity to regions on both sides of the site of a DSB target by Cas9. These blocks flank a selection cassette consisting of the *dhfr* selectable marker, a promoter from the *cam* gene, and a transcriptional terminator from the *hrp* gene. (C) The predicted result from HR-mediated repair. (D) Diagram showing the diagnostic PCRs used to confirm the products of HR and integration of the *dhfr* cassette into the genome. Diagrams 1–3 show integration into genes on chromosome 12, while 4 shows integration into an unlinked *var* gene on chromosome 6. (E) Gel electrophoresis of PCR products shown in D. *acs7*, acyl-CoA synthetase 7; *cam*, calmodulin; Cas9, CRISPR-associated protein-9 nuclease; *dhfr*, dihydrofolate reductase; DSB, double-strand break; FIKK, phenylalanine-isoleucine-lysine-lysine-motif–containing kinase; hb, homology block; HR, homologous recombination; *hrp*, histidine-rich protein 2; PCR, polymerase chain reaction; TARE, telomere-associated repeat element.

In a previously published study, we utilized several rounds of X-ray irradiation to generate random DNA DSBs within the genome of a recently cloned, cultured line of the NF54 isolate of *P*. *falciparum* [18]. This allowed us to observe the products of DNA DSB repair generated by the parasite in response to this damage. We found that within subtelomeric chromosomal regions, parasites used a combination of telomere healing and repair by HR to preserve genome integrity. We also observed the creation of a chimeric *var* gene; however, given the inexact nature of generating random DNA breaks using repeated rounds of X-ray irradiation, it was not possible to precisely determine the sequence of events that led to the genomic changes that were ultimately defined. To examine the process of DSB repair within subtelomeric regions more accurately, in the current study, we generated precisely defined breaks using Clustered Regularly Interspaced Short Palindromic Repeats/CRISPR-associated protein-9 nuclease (CRISPR/Cas9) within a subtelomeric region at the “left” end of chromosome 12. This region was selected since it appears to be fully intact, containing a large complement of variant-antigen–encoding genes (3 *var* genes and 3 *rifin* genes) and an approximately 15-kb array of TAREs extending to the telomeric repeats. Specific guide RNAs were designed to target DSBs to sequences upstream of the coding regions of two of the *var* genes (Pf3D7_1200400 and Pf3D7_1200600) and a *rifin* gene (Pf3D7_1200500) within this subtelomeric domain (Fig 1A). In each case, in addition to the Cas9 expression construct, co-transfection of an episomal plasmid provided a suitable template for repair by HR as well as the guide RNA expression cassette. This repair template included DNA sequences identical to sequences flanking the site of the break and a drug selectable cassette that enabled us to detect repair by HR (Fig 1B).

Each construct was transfected into cultured parasites and several individual subclones obtained. Successful break and repair was determined by polymerase chain reaction (PCR) amplification across the site of the break, followed by sequencing of the amplified product. In all cases, DNA repair appeared to have occurred by HR, yielding the precise products predicted from crossover events within the sequences of shared identity between the plasmid template and the chromosome (Fig 1D and 1E). These repair events are typical of those previously observed in numerous studies that have employed CRISPR/Cas9 for genome editing and reflect the apparent tendency of malaria parasites to almost exclusively use HR for DSB repair [19,20]. Similar HR-mediated recombination was observed for a DSB targeted to Pf3D7_0617400, a *var* gene within an internal cluster on chromosome 6 (Fig 1D and 1E). Thus, DSBs within *var*-gene–containing regions of the genome are readily repaired by HR with an apparent efficiency similar to other regions of the genome. The genome of one of the clones in which the DSB was targeted upstream of Pf3D7_1200600 was also fully sequenced using single-molecule, long-read sequencing technology and displayed no detectable alterations apart from the product of repair at the site of the Cas9-targeted break (all genome sequence data are available at the National Library of Medicine BioProject database, accession PRJNA515738, sample VAR2CAL). This indicates that simply inducing DNA repair by HR within a subtelomeric region does not result in any unusual repair products or cause instability throughout the rest of the genome.

### Telomere healing can occur at various positions with respect to a DSB to stabilize a broken chromosome end

**Fig 2:**
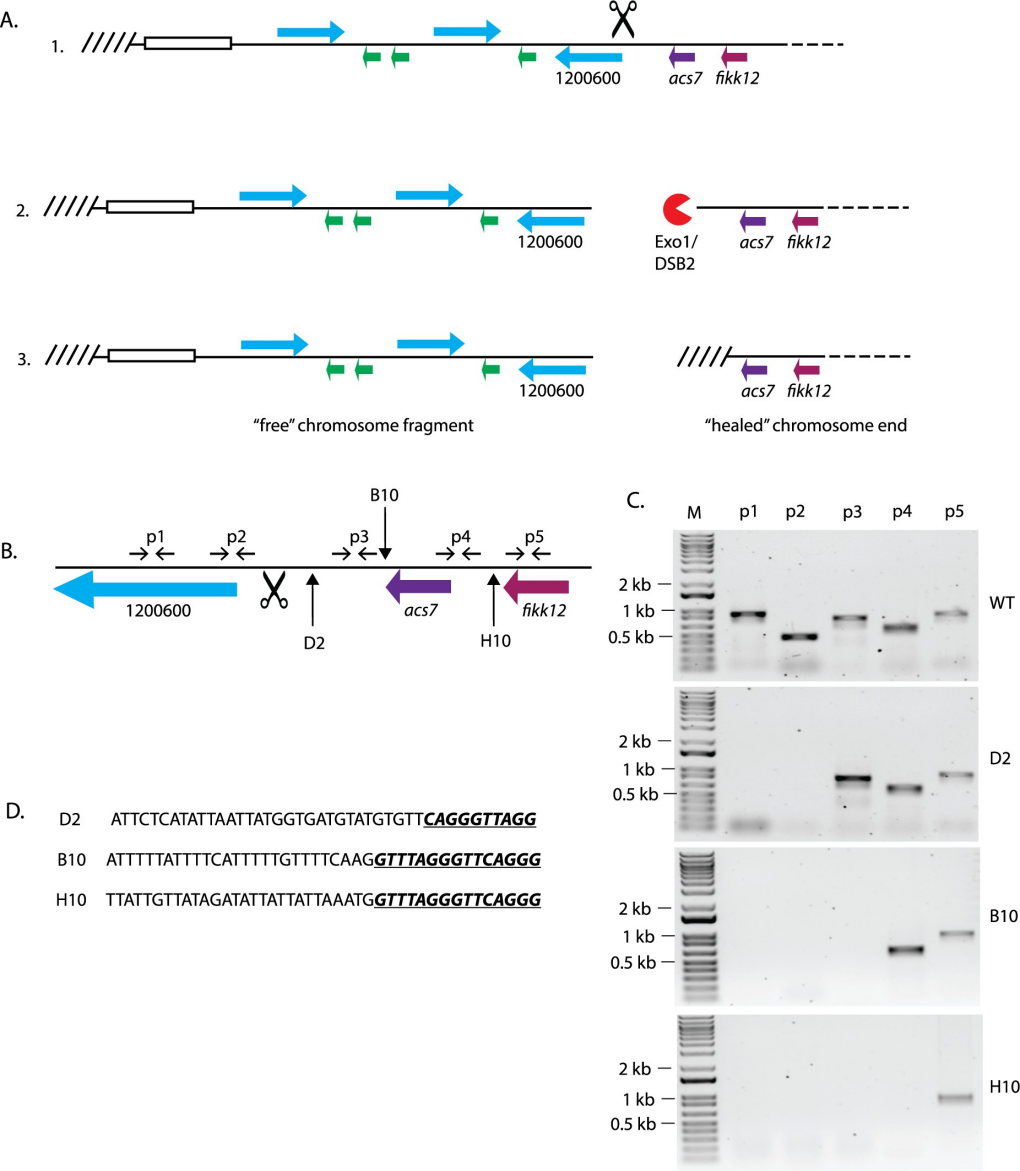
Telomere healing events resulting from DSBs within a subtelomeric region of chromosome 12. (A) The subtelomeric region of the “left” end of chromosome 12 is shown (1), and the targeted site for a break by Cas9 is shown by scissors. This site is between *var* gene PF3D7_1200600 and the gene *acs7*. After induction of the DSB (2), an exonuclease (Exo1/DSB2) resects the DNA end. Telomerase then “heals” the end through the addition of telomere repeats (3), resulting in a stabilized chromosome end and a free DNA fragment. (B) An enlarged schematic of the chromosome region surrounding the *acs7* gene. The sites of telomere healing in the clones D2, B10, and H10 are shown by vertical arrows, and the site of the targeted DSB is shown by scissors. The position of PCR primers used to detect DNA resection are shown as paired horizontal arrows. (C) PCR products from reactions using each primer pair shown in B for the parasite clones D2, B10, and H10. (D) Sequencing across the site of telomere healing shows the addition of telomere repeat sequences (underlined italics) to unique positions in each clone. *acs7*, acyl-CoA synthetase 7; Cas9, CRISPR-associated protein-9 nuclease; DSB, double-strand break; Exo, exonuclease; FIKK, phenylalanine-isoleucine-lysine-lysine-motif–containing kinase; PCR, polymerase chain reaction.

During asexual replication in RBCs, parasites are haploid and thus only possess an exact homolog for DSB repair by HR during the relatively brief time after DNA replication but prior to nuclear division. During the remainder of the replicative cycle, no direct homologs for repair are available, and therefore DSB repair typically occurs either through recombination with regions of sequence similarity elsewhere in the genome or, when breaks occur near the ends of chromosomes, through telomere healing. Our previous study on the repair of random breaks generated by exposure to X-rays detected frequent telomere healing events within subtelomeric regions [18]. Analyses of the repair products were consistent with an established model for telomere healing in which after detection of a DSB, an exonuclease resects the chromosome end until a 3′ single-stranded sequence is revealed that has homology to telomerase RNA. This allows priming by the RNA and initiation of telomere repeat addition by telomerase, resulting in the addition of a telomere and completion of the healing event (Fig 2A). However, given the random nature of breaks caused by X-rays, it was not possible to know the site of the DSB, and thus the process of resection leading to telomere addition could only be inferred. In addition, it was not possible to determine whether there are preferred sites of telomere addition or whether this process can occur at various positions within a stretch of the chromosome.

To more closely examine how telomere healing occurs in *P*. *falciparum*, we used the CRISPR/Cas9 system described above to target a DSB to the noncoding sequence between *var* gene Pf3D7_1200600 and the adjacent *acs7* gene (Pf3D7_1200700) (Fig 2A). This site sits near the boundary between the subtelomeric region and the core of chromosome 12 and is unique within the genome, thus for HR to be used for repair, we anticipated that a template must be provided. Consistent with this hypothesis, when provided with a template for repair, a break at this site is efficiently repaired by HR (Fig 1). In contrast, when we provided no template for repair, we only detected telomere healing events. Specifically, 9 clones isolated from the transfected population were analyzed by PCR using primers to amplify sequences on both sides of the DSB (Fig 2B). Loss of DNA segments as a result of DNA resection prior to the addition of telomere repeats is a hallmark of telomere healing. These reactions allowed us to map the extent of resection in each clone. Precise mapping of the site of telomere repeat addition was then performed by PCR using a primer specific for the conserved telomere repeat sequence and a primer near the mapped deletion followed by sequencing of the amplified product. From these 9 clones, three different positions of telomere healing were identified as exemplified by clones D2, B10, and H10 (Fig 2C), indicating that during resection, telomerase can initiate the addition of telomere repeats at several different positions within this region of the chromosome. The sequences at the site of telomere repeat addition shared no obvious similarity to each other, therefore it was not possible to derive any conclusions for why healing events occur in any particular position. However, previous work from several groups have identified putative secondary structures, including hairpins and G-quadruplexes, that can influence DNA stability and occur frequently within subtelomeric regions in *P*. *falciparum* [21–24]. It is possible that the existence of such structures could affect the rate of DNA resection or telomerase recruitment, thereby influencing the site of healing even when not found directly at the position where the repeats are added.

### Free chromosome fragments generated by telomere healing are highly recombinogenic

Telomere healing is an efficient mechanism for stabilizing a chromosome when a DSB occurs near its terminus and thus is an effective way to maintain genome integrity. However, the chromosome fragment between the site of the break and the original telomere repeats is presumed lost as a result of the healing event (Fig 2A). The typical subtelomeric structure, including variant-antigen–encoding genes and TAREs, can be regenerated through subsequent telomere conversion events; however, the loss of the original subtelomeric region, including any resident variant-antigen–encoding genes, would represent a reduction in overall sequence diversity. Thus, repair of chromosome breaks by telomere healing would lead to homogenization rather than diversification of the parasite’s variant antigen repertoire. Alternatively, given that broken DNA ends can be highly recombinogenic, the released DNA fragment could serve as a template for additional recombination events at other sites in the genome. If so, telomere healing could instead act as a driving force for the diversification of genes unlinked to the site of the original DSB. To investigate this possibility, we aimed to determine the fate of the free chromosome fragment between a site of telomere healing and the original chromosome end. The free chromosome fragment liberated by the healing events between *var* gene Pf3D7_1200600 and *acs7* on chromosome 12 described above (Fig 2A) is ideal for this analysis because of its length and the number of genes within this region, thus providing numerous potential sequences for recombination.

**Fig 3:**
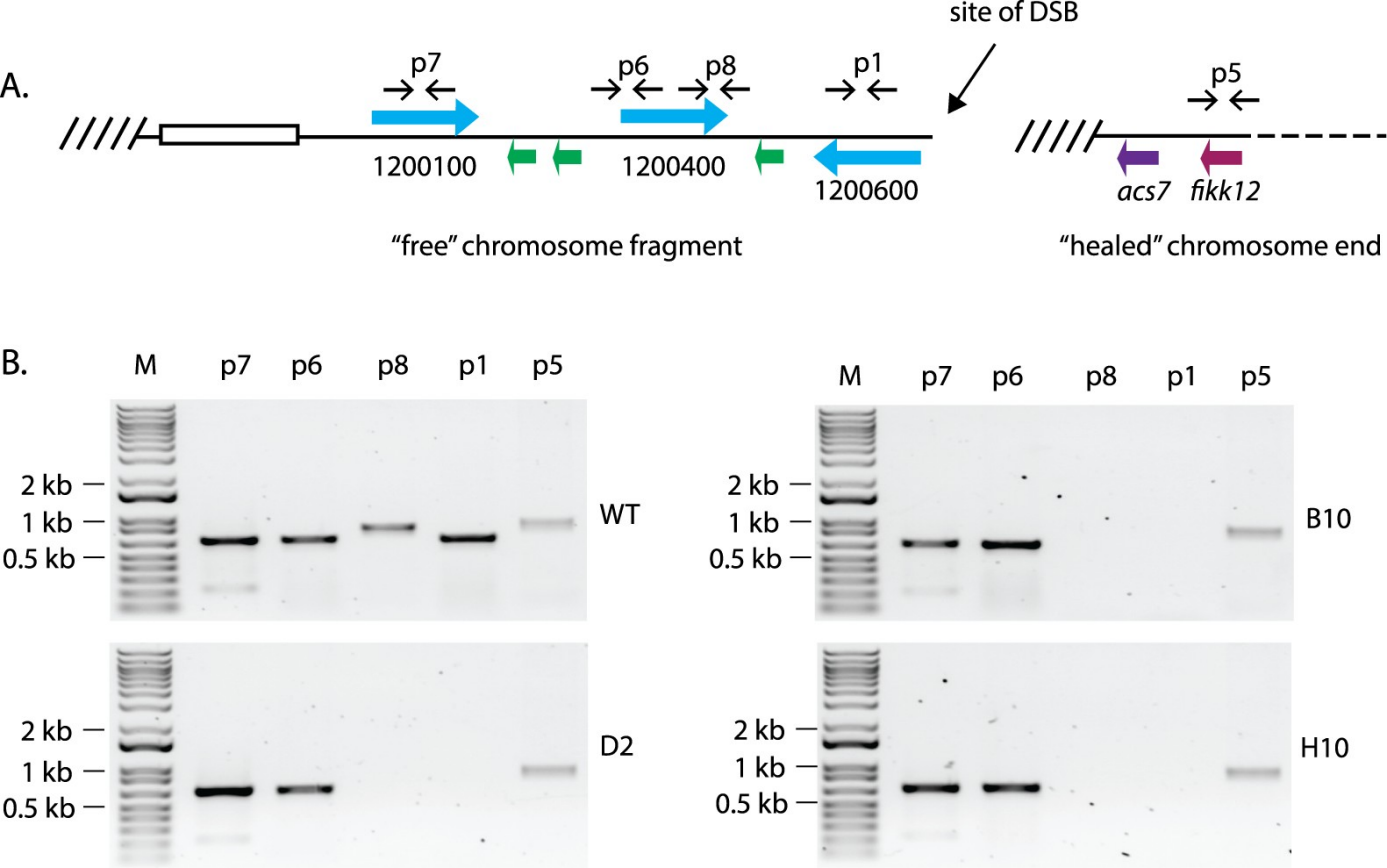
Detection of DNA fragments after truncation of the end of chromosome 12. (A) Diagram of the “left” end of chromosome 12. The annotation numbers for *var* genes are shown. TAREs are shown as an open box, and telomere repeats are shown as hashed lines. The positions of specific PCR primers used to amplify each gene are shown as paired horizontal arrows. The site of the DSB induced by Cas9 expression is shown. (B) Products of PCR amplifications to detect each gene, demonstrating that several portions of the chromosome persist in the genome despite the truncation of this end of chromosome 12. *acs7*, acyl-CoA synthetase 7; Cas9, CRISPR-associated protein-9 nuclease; DSB, double-strand break; FIKK, phenylalanine-isoleucine-lysine-lysine-motif–containing kinase; PCR, polymerase chain reaction; TARE, telomere-associated repeat element.

As a preliminary assessment of the fate of this chromosome fragment, we used genomic DNA from the 3 clonal lines that displayed independent telomere healing events and performed PCR with primers specific to each gene within this region (Fig 3A). If this chromosome fragment had been lost during the telomere healing event, all the reactions should have failed to amplify a product. However, we instead observed consistent amplification of several genomic segments (Fig 3B), suggesting that these sequences persisted within the genomes of these parasite lines. The *var* gene Pf3D7_1200400 appeared to have been divided, with its 5′ end still detectable by PCR while the 3′ end was apparently lost. Sequencing of the PCR products verified their identity and ruled out the possibility of nonspecific amplification. However, given that our previous experiments indicated that this end of chromosome 12 was truncated, it was not clear where in the genome these gene segments now resided.

To more precisely identify changes in the genomes of these 3 parasite lines, we employed single-molecule, long-read whole-genome sequencing technologies (sequence data available at the National Library of Medicine BioProject database, accession PRJNA515738, samples D2, B10, and H10). These technologies generate individual reads of greater than 10 kb, thereby enabling us to easily identify structural changes in the genome, including recombinations, deletions, duplications, and gene conversion events. For each of the lines analyzed, the genome assembly precisely detected the telomere healing events previously mapped by PCR without ambiguity, providing independent verification of these events. We then examined the entire genome assemblies to determine the fate of the other sequences within the original subtelomeric region adjacent to the site of the break.

**Fig 4:**
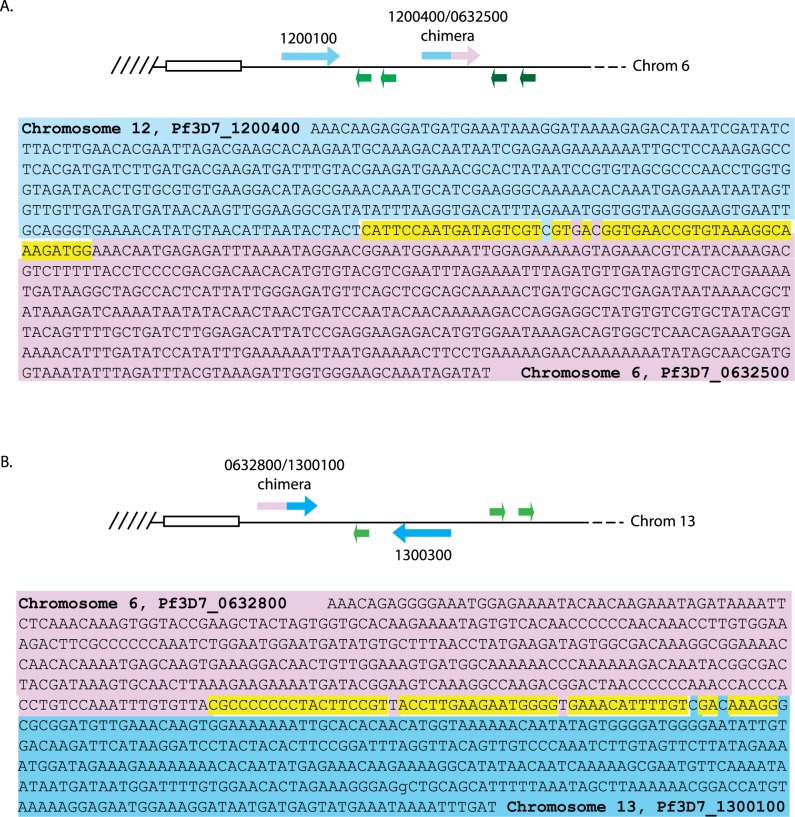
Sequences of chimeric *var* genes identified by whole-genome sequencing and verified by PCR amplification. (A) Top: schematic diagram of one end of chromosome 6 after recombination. Bottom: chimeric gene consisting of sequences from Pf3D7_1200400 (blue) and Pf3D7_0632500 (pink). (B) Top: schematic diagram of one end of chromosome 13 after recombination. Bottom: chimeric gene consisting of sequences from Pf3D7_0632800 (pink) and Pf3D7_1200100 (blue). Bases that are identical between the two genes are highlighted in yellow. PCR, polymerase chain reaction.

Despite having distinct sites of telomere healing, the patterns of PCR amplifications were identical in the 3 lines analyzed (Fig 3B), suggesting that the released DNA fragment had undergone similar or identical recombination events in each of these clones. In these lines, searches of the genome assemblies for the gene fragments that were detectable by PCR but were predicted to have been lost as a result of telomere healing identified two new, chimeric *var* genes that appeared to be the result of recombination events. In each case, we detected a newly generated chimeric *var* gene comprised of Pf3D7_1200400 (5′ end) and Pf3D7_0632500 (3′ end) as well as a second chimera of Pf3D7_0632800 (5′ end) and Pf3D7_1300100 (3′ end) (Fig 4). Both of these recombination events created chimeric *var* genes with intact open reading frames and thus are presumably functional genes. Gene-specific PCR across the sites of recombination followed by sequencing of the PCR products verified the existence of these chimeric *var* genes.

### A sequential cascade of recombination events is predicted to result in the creation of chimeric *var* genes

In the examination of the genome sequences from the recombinant parasite lines, not only did we detect recombination events involving genes near the site of the targeted DSB, we also found that recombination had occurred between genes on different chromosomes, completely unlinked to the site of the break. For example, we detected a chimeric *var* gene resulting from a recombination between Pf3D7_0632800 on chromosome 6 and Pf3D7_1300100 on chromosome 13. How does repair of a DSB on chromosome 12 lead to this distant recombination event? By considering the different chimeric sequences and presuming that HR is the dominant mechanism of repair, we can deduce the likely sequence of events that resulted in the generation of the chimeric *var* genes in these lines.

**Fig 5:**
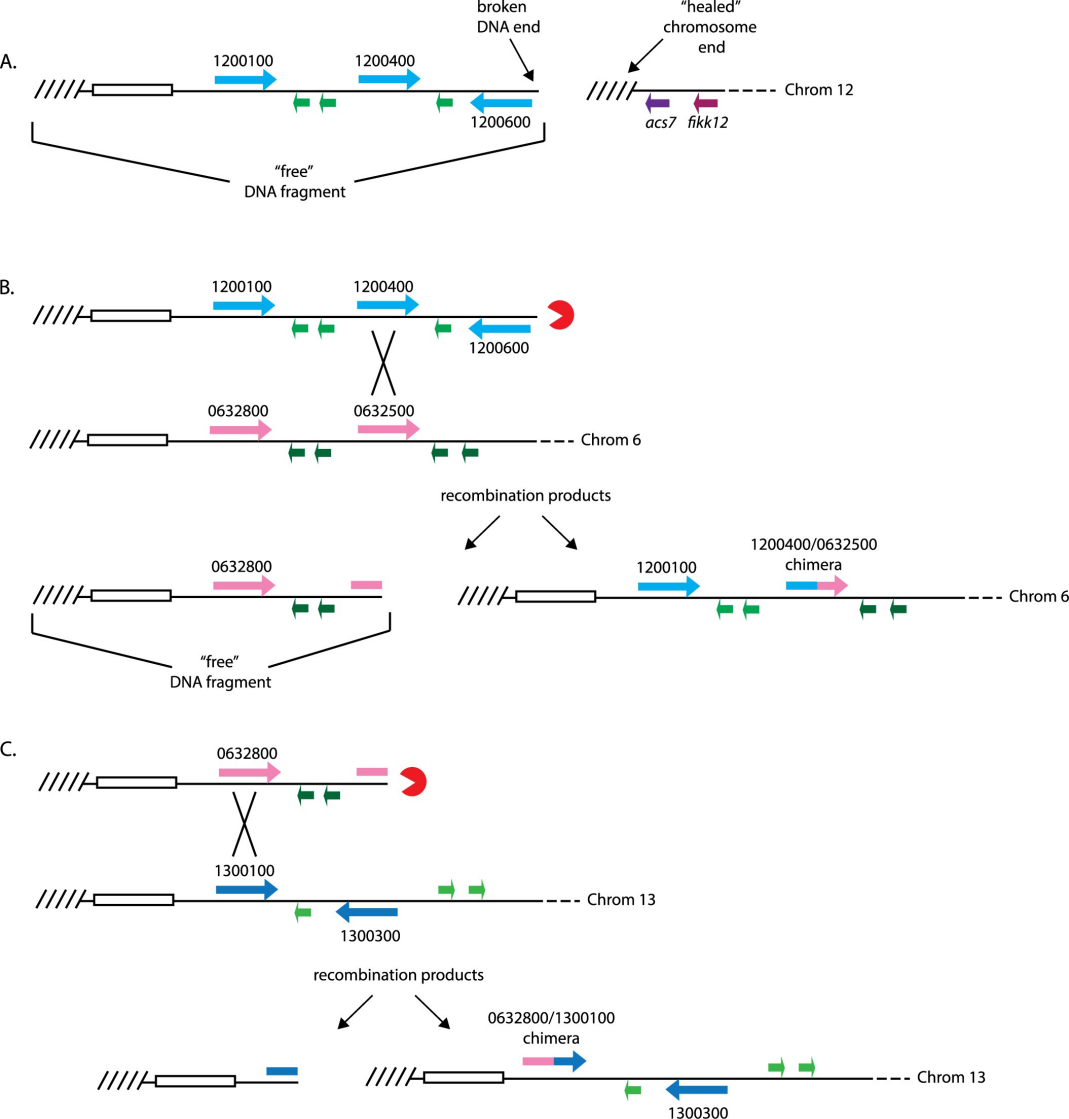
Sequential recombination events initiated by telomere healing that lead to the creation of chimeric *var* genes. (A) Diagram of the “left” end of chromosome 12 after induction of a DSB between *var* gene PF3D7_1200600 and *acs7*. The annotation numbers for *var* genes are shown. TAREs are shown as an open box, and telomere repeats are shown as hashed lines. Note that new telomere repeats are now found directly downstream of *acs7*, creating a new end of chromosome 12 and liberating the remainder of the chromosome as a free DNA fragment. (B) The free DNA fragment derived from the end of chromosome 12 is shown aligned with the end of chromosome 6. Exonuclease resection of the free DNA end is shown by a Pac-Man symbol. Recombination between *var* genes PF3D7_1200400 and PF3D7_0632500 is shown with an X. Two products of recombination are shown, the new end of chromosome 6 including the chimeric *var* gene created by the recombination (right) and the new free DNA fragment (left). C) The free DNA fragment derived from the end of chromosome 6 is shown aligned with the end of chromosome 13. Exonuclease resection of the free DNA end is shown by a Pac-Man symbol. Recombination between *var* genes PF3D7_0632800 and PF3D7_1300100 is shown with an X. Two products of recombination are shown, the new end of chromosome 13 including the chimeric *var* gene created by the recombination (right) and the new free DNA fragment (left). *acs7*, acyl-CoA synthetase 7; Chrom, chromosome; FIKK, phenylalanine-isoleucine-lysine-lysine-motif–containing kinase; TARE, telomere-associated repeat gene.

Both targeted PCR and whole-genome sequencing indicate that the initial CRISPR/Cas9 induced DSB was repaired by a telomere healing event between *var* gene Pf3D7_1200600 and *acs7*. This stabilized the end of chromosome 12 but liberated a free DNA fragment that included 3 *var* genes and 3 *rifin* genes (Fig 5A). This DNA fragment then underwent a recombination between one of these *var* genes (Pf3D7_1200400) and a *var* gene on chromosome 6 (Pf3D7_0632500), creating the observed Pf3D7_1200400/Pf3D7_0632500 chimera and leading to the transposition of the original end of chromosome 12 into chromosome 6 (Fig 5B). The genome assemblies place the *var* gene Pf3D7_1200100 and *rifin* genes Pf3D7_1200200 and Pf3D7_1200300 on chromosome 6, verifying this event. While this event stabilized the original free DNA fragment, it also liberated the end of chromosome 6, thus generating a new free DNA fragment that includes a *var* gene (Pf3D7_0632800) and 2 *rifin* genes (Pf3D7_0632600 and Pf3D7_0632700). This DNA fragment underwent a recombination event between Pf3D7_0632800 and a *var* gene on chromosome 13 (Pf3D7_1300100), creating the observed chimera and replacing the end of 13 (Fig 5C). The free DNA fragment generated by this event consists primarily of the highly repetitive TAREs and telomere repeats that are found at most chromosome ends; therefore, it is not possible to determine whether it was subject to additional recombination events.

### Recombination events between *var* genes are not random

Previous analysis of ectopic recombination between *var* genes indicated that these events are not random but rather display a structured pattern [3]. We similarly observed that certain recombination events appear to be favored. For example, all 3 clonal lines analyzed displayed identical recombination patterns, suggesting that these events are favored when a DSB occurs within this region of the genome. The sites of telomere healing varied in these clones (Fig 2B), indicating that these lines are derived from truly independent initial break and repair events. To confirm this conclusion, we introduced a DSB into the same chromosomal region using the CRISPR/Cas9 construct shown in Fig 2 in an alternative subclone of NF54 called DC-J [25]. Analysis of the resulting parasite population by both PCR and genome sequencing identified the same recombination cascade described in Fig 5 (sequence data available at National Library of Medicine BioProject database, accession PRJNA515738, sample RIF-FLP-DCJ). Examination of the sites of recombination that resulted in the chimeric *var* genes identified regions of approximately 95% sequence identity of approximately 50 bp (Fig 4), consistent with the now well-established conclusion that HR is the dominant repair pathway used by these parasites. Thus, it seems likely that DSBs introduced into other regions of the genome would result in alternative recombination cascades.

Given the dominant role of HR in repairing DSBs in malaria parasites and its dependence on stretches of sequence identity for recombination, it is possible that the cascade shown in Fig 5 is the only sequence of events that can result from a DSB at this position of chromosome 12. To determine whether alternative, low-probability recombinations might have also occurred, we isolated genomic DNA from an uncloned population of transfected parasites in which a DSB was targeted to the subtelomeric region of chromosome 12 and subjected it to long-read, whole-genome sequencing. Analysis of the genome sequences from this population identified the expected telomeric healing event and the same recombination cascade observed in D2, B10, and H10; however, we also detected an additional chimeric *var* gene distant from the site of the DSB, a chimera of *var* genes Pf3D7_1300100 and Pf3D7_0900100 (sequence data available at the National Library of Medicine BioProject database, accession PRJNA515738, sample 700CAL-WT). We did not detect this chimeric sequence from genome assemblies obtained from parasites in which we did not induce a DSB, suggesting that it similarly is a product of recombination events induced by the initial Cas9-targeted DSB. However, we were unable to identify additional events linking the creation of this chimeric gene to the original break, and thus it is possible that this chimeric *var* gene was generated independently.

### Evidence for recombination cascades within other chromosomal regions

**Fig 6:**
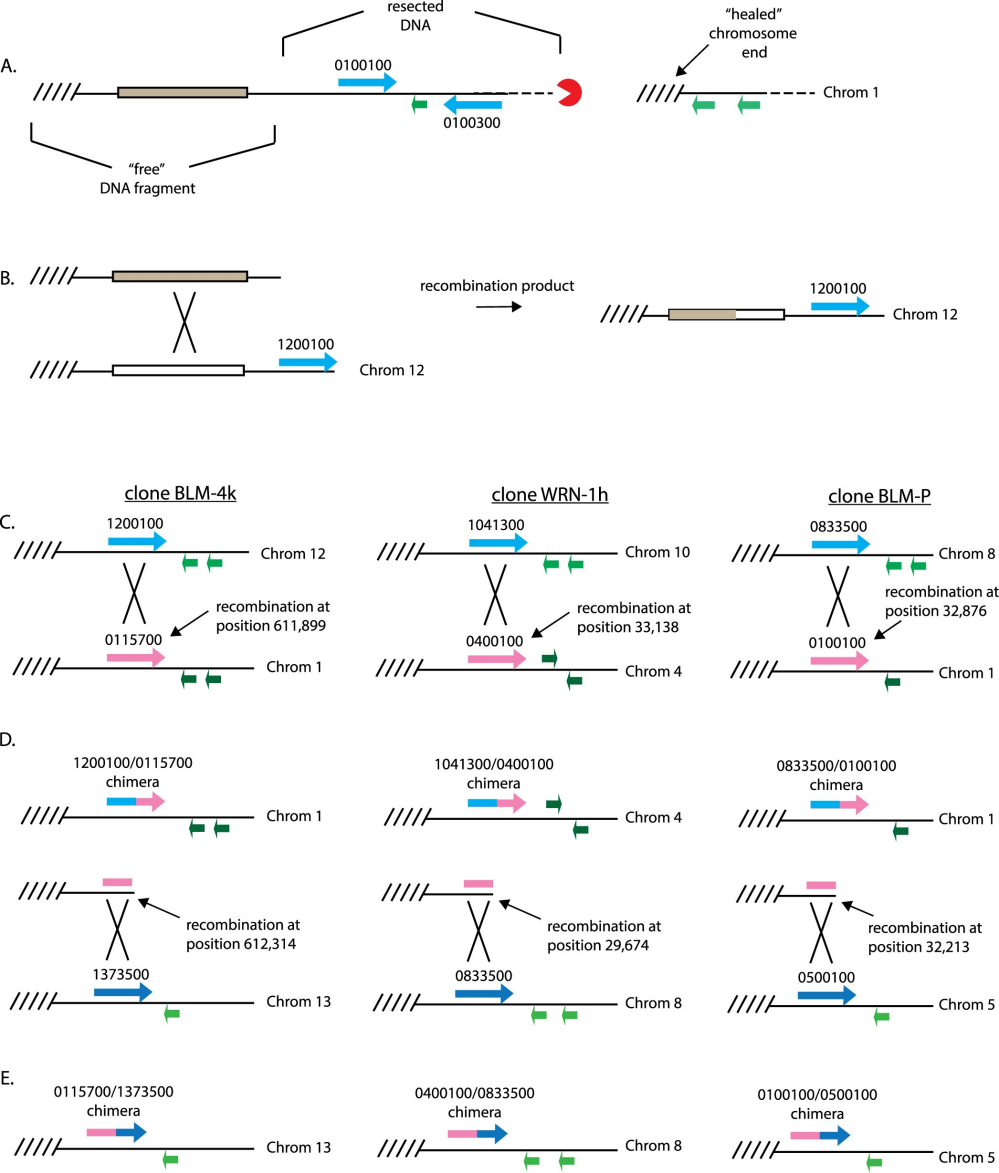
Sequential recombination events between subtelomeric regions at additional genomic loci. Previous work [18] identified a telomere healing event on chromosome 1 (A) that resulted in the translocation of the released subtelomeric fragment into a position near the end of chromosome 12 (B). Spontaneous sequential recombination events were also detected in subcloned parasite lines isolated by Claessens and colleagues [23] (C–D). Whole-genome sequencing of subcloned lines of 3D7 parasites identified multiple recombination events within *var* genes. For 3 subclones (named BLM-4k, WRN-1h, and BLM-P) closely spaced recombination events were suggestive of sequential cascades of recombination leading to multiple chimeric *var* genes. The putative order of events was inferred based on the position of the recombinations with reference to the central *var* gene within each cascade (pink). The initial recombination event for each clone occurred between 2 *var* genes near the chromosome ends (C), creating a chimeric *var* gene and releasing a free DNA fragment (D). Recombination of the free DNA fragment with a *var* gene at another chromosome site resulted in the creation of an additional chimeric *var* gene (E). Detection of these chimeric *var* genes was originally reported in supplemental data table S1 of reference [23]. Chrom, chromosome.

Our discovery of a sequential cascade of recombination leading to the creation of chimeric *var* genes resulted from the generation of targeted DSBs within a subtelomeric region of chromosome 12. This recombination cascade appears to be highly reproducible, leading us to question how common similar events are at other regions of the genome and, perhaps more importantly, whether such recombinatorial cascades can be detected in naturally replicating parasites. Upon re-examination of the telomere healing events we previously described [18], we find evidence for a similar recombination that was likely initiated by the release of a free DNA fragment during repair of the chromosome end. Specifically, a telomere healing event resulted in the truncation of approximately 90 kb from the end of chromosome 1. However, rather than being lost, a 13,886 bp fragment of the truncated region was found integrated into a subtelomeric region of chromosome 12 (Fig 6A and 6B), indicative of the type of recombinations linked to telomere healing described in Fig 5. This event did not involve *var* genes, suggesting that this mechanism is not exclusive to *var* genes in particular or coding regions in general and instead simply reflects the DNA repair mechanisms that are active in the parasites.

The event described above likely resulted from a DSB induced by exposure to X-ray irradiation. We were curious if similar recombination events could result from spontaneous breaks that occur during normal parasite replication. Several recent papers have described the analysis of clonal parasite lines isolated after extensive growth in culture. Some of these lines displayed multiple recombination events resulting in the creation of several chimeric *var* genes [17,23,26], suggesting the possibility of recombination cascades. No detailed mechanism was identified for the origins of the chimeric genes in these clones; however, Claessens and colleagues [23] noted that they tended to observe the occurrence of ectopic recombination events in clusters; specifically, that a translocation between 2 subtelomeric *var* genes highly increased the chance of detecting additional recombination events nearby. Given that such clustered recombination events could result from the type of recombination cascade we observed on chromosome 12, we re-examined the detailed data set provided by Claessens and colleagues to determine whether any of the recombination patterns they reported are consistent with a cascade mechanism. While several clones displayed multiple recombination events that could have occurred sequentially, 3 examples are of particular interest and shown schematically in Fig 6C–6E. Each shows a two-step cascade involving 3 *var* genes and leading to the creation of 2 *var* gene chimeras. In each case, the central *var* gene within the recombination cascade has undergone two closely spaced recombinations, with the positions of the recombinations providing the likely order of events as the cascade moves toward the chromosome end. The genome sequence data sets for these clones were generated from 100-bp paired-end reads, from which it is difficult to assemble the highly repetitive sequences near and within telomeres; thus, telomere healing events would have been difficult to detect with confidence. It is therefore not possible to know if the putative recombination cascades shown in Fig 6C–6E were initiated by telomere healing; nonetheless, a sequential cascade of recombinations similar to what we describe in Fig 5 seems highly likely. The recombination events detected in these clones did not involve any of the *var* genes that underwent recombination after we induced a DSB on chromosome 12, suggesting that this recombinatorial mechanism can likely occur at any subtelomeric region of the parasite’s genome.

## Discussion

The data presented here describe how a single DSB within the subtelomeric regions of *P*. *falciparum* chromosomes can stimulate a sequence of recombination events between *var* genes, thus accelerating the generation of diversity within this gene family. Two key aspects of *Plasmodium* DNA repair contribute to this phenomenon: 1) near-complete dependence on HR to repair DSBs and 2) a propensity to use telomere healing when breaks occur with subtelomeric regions. The dependence on HR results from the parasite’s inability to use classical nonhomologous end joining (NHEJ) to repair DSBs because of the evolutionary loss of the enzymes required for this pathway [27]. This ensures that all DSB repair events will involve recombination between similar sequences, and thus when breaks occur near or within *var* genes, chimeric *var* genes will be the dominant products of repair. Such chimeric genes are likely to maintain intact open reading frames as well as the basic domain structures of the encoded proteins, characteristics observed previously during the generation of chimeric *var* genes [16,17,21].

The evolutionary loss of the NHEJ pathway resulting in near-complete dependence on HR for DSB repair was initially puzzling [28], particularly given that these parasites exist primarily as haploid organisms and thus generally do not possess a template for repair. However, extensive use of NHEJ to repair DSBs within subtelomeric regions would not only reduce the frequency of the generation of chimeric *var* genes, it would also lead to the creation of pseudogenes through the frequent introduction of frame shifts and nonsense mutations. Thus, the loss of the NHEJ pathway ensures the use of HR when breaks occur near *var* genes, thereby driving the diversification of this gene family. Given the importance of antigenic variation in the lifestyle of malaria parasites, this likely provided the selective advantage that led to the evolutionary loss of NHEJ. This is consistent with the dominance of NHEJ for DSB repair in the closely related parasite *Toxoplasma gondii* [29,30], which does not undergo antigenic variation during infection and thus would not benefit from frequent ectopic recombination events.

The role of telomere healing in driving *var* gene diversification results from its generation of highly recombinogenic free chromosome fragments, the first step in a sequential recombination cascade that can ultimately result in the creation of multiple chimeric *var* genes. Telomere healing both stabilizes the broken chromosome end and generates a free chromosome fragment that can be resected to reveal sequences that share stretches of identity with subtelomeric regions on other chromosomes. Recombination between such regions not only results in chimeric sequences, it also generates a new free DNA chromosome fragment that can undergo an additional round of resection and recombination. Thus, telomere healing has the potential to initiate a sequential cascade of recombination events that can result in the creation of chimeric genes completely unlinked to the original DSB. Telomere healing is frequently observed in cultured lines of *P*. *falciparum* [18,31–33], and evidence indicates that it is a common occurrence in the field as well [34–37], suggesting that it likely contributes significantly to the generation of sequence diversity in natural parasite populations.

In contrast, one of the first ectopic recombination events described that created a chimeric *var* gene involved the transposition of a subtelomeric region from one chromosome to another in the apparent absence of healing [38]. This recombination event led to the duplication of one subtelomeric region and the loss of another. It is possible that the displaced subtelomeric region could have initiated a recombination cascade similar to what we describe here; however, this was not investigated. Thus, it is possible that sequential recombination in the absence of telomere healing could also contribute to *var* gene diversification. Work in human cancer cells has similarly shown that displacement or transposition of chromosome fragments containing telomeres can result in cascades of recombination involving several chromosome ends [39]. In principle, any chromosome break or product of repair, including products of nonreplicative transposition or break-induced repair, that generates an unstable free DNA fragment can initiate additional recombination events throughout the genome [40,41]. These examples from cancer cell lines are consistent with the model we have proposed for cascades of *var* gene recombination in malaria parasites, suggesting that this process is evolutionarily conserved.

Left unaddressed by the work presented here is the source of the initial DSB that can lead to telomere healing and a cascade of recombination. It is possible that the subtelomeric regions have a rate of DSB formation that is similar to the rest of the genome but that such breaks are often lethal when they occur in the central chromosomal regions and thus are not observed. Alternatively, several studies have suggested that subtelomeric regions are uniquely susceptible to DSB formation, thus driving the process of recombination and generating diversity at an accelerated rate. These studies have described structural elements found within *var* genes and subtelomeric regions that could serve as a source of DSBs, including secondary structures [21] and G-quadruplexes [22,23]. The concept of hyper-recombinogenic subtelomeric regions is becoming appreciated as a major source of genetic variation in organisms ranging from other protozoans [42] to yeast [43] to plants [44], and it has been proposed that “mild telomere dysfunction” can serve as a significant source of genetic variation driving adaptive evolution [45]. Interestingly, for yeast, it was proposed that sources of telomere instability that favor repair by HR rather than NHEJ would be more likely to drive adaptive evolution [43], a concept consistent with the proposal that loss of classical NHEJ in malaria parasites provides a selective advantage by facilitating increased rates of subtelomeric recombination.

Previous work described structured rearrangements between *var* genes resulting from recombination events that were “error-free” and maintained the domain architecture of the encoded PfEMP1 [16,17]. Comparisons of *var* gene sequences and their chromosomal location also found that *var* genes preferentially recombine with other *var* genes located within the same chromosomal position and oriented in the same direction with respect to the chromosome end [46]. This recombinational preference is thought to have led to the divergence of type A, B, and C *var* genes [5,6]. Additional conditions—for example, the alignment of subtelomeric regions within clusters near the nuclear periphery [47], thus bringing specific subsets of *var* genes into close proximity—might also influence potential recombination events, thereby leading to the structured nature of the recombinations that are observed. These influences appear to be significant because in our study, we observed identical recombination events occurring multiple times independently. Further, BLAST searches identified the stretch of sequence of homology surrounding the site of recombination shown in Fig 4B within *var* genes on chromosomes 6, 7, 8, 12, and 13; however, we only ever detected recombination between the genes on chromosomes 6 and 13, suggesting the influence of additional constraints.

The cumulative data provided by multiple studies therefore describe a system in which the loss of NHEJ combined with the concentration of variant-antigen–encoding genes within subtelomeric regions leads to the continuous generation of sequence diversity within these gene families. The reliance on HR in the absence of NHEJ limits the number of possible recombination events, thereby preserving the basic architecture of the encoded proteins. This system therefore achieves a balance between the generation of antigenic diversity with constraints on protein structure and function, enabling parasites to maintain chronic infections and efficient transmission between hosts with varying degrees of pre-existing immunity.

## Materials and methods

### Ethics statement

This study uses no human or animal subjects. Human RBCs are used solely to propagate parasites in culture and are obtained from surplus supplies from a commercial source. Weill Cornell Medical College and the NIH have concluded that this does not constitute human subject research and therefore does not require IRB approval.

### Culture and genetic manipulation of parasites

All parasite lines are derivatives of the *P*. *falciparum* isolate NF54 and are isogenic with the reference strain 3D7. Parasites were reared in a standard culture system at 5% hematocrit in media containing RPMI 1640 (Corning Life Sciences, Tewksbury, MA, USA), 0.5% Albumax II (Invitrogen, Carlsbad, CA, USA), 0.25% sodium bicarbonate, and 0.1 mg/ml gentamicin in an atmosphere of 5% oxygen, 5% carbon dioxide, and 90% nitrogen. Clonal parasite lines were obtained by limiting dilution [48]. Parasites were transfected by electroporation [49,50] using derivatives of the plasmids pL-6_eGFP and pUF1_Cas9 for CRISPR/Cas9-based genome editing as described by Ghorbal and colleagues [19]. The guide RNAs for targeting DSBs to specific genomic positions were 5′-TAAGTATATAATATTGTCAGACGCACGCGTCTTTTTTAGAGCTAGAAA-3′ for Pf3D7_1200400; 5′-TAAGTATATAATATTGTATTTAATACTTATGTCACATTTTAGAGCTAGAAA-3′ for Pf3D7_1200500; 5′-TAAGTATATAATATTGTGGAATAAAGAAACATACTTTTTTTTAGAGCTAGAAA-3′ for Pf3D7_1200600; and 5′-TAAGTATATAATATTGTATGTAGTGGTTGCTGTCCTTTTTAGAGCTAGAAA-3′ for Pf3D7_0617400. Homology blocks for genome modification were amplified from parasite genomic DNA by PCR and inserted into pL-6_eGFP by infusion cloning (Clontech, Takara Bio USA, Mountain View, CA, USA). Primer pairs for amplifying specific homology blocks were 5′-AATACTCGCGGCCGCACTAGTGCACTCTGGCAACCAGC-3′ and 5′-AAGACAGATCTTCGGACTAGTCGATCGACAAGCGTCGC-3′ for Pf3D7_1200400 homology block 1; 5′-TATTTATTAAATCTAGAATTCGGATATGATTGGAGAAGTTGTAC-3′ and 5′-ATTATTTTTACCGTTCCATGGCCACCAATCTTCTCGTAATTG-3′ for Pf3D7_1200400 homology block 2; 5′-AATACTCGCGGCCGCACTAGTCTGTAATTTCAGCAGTAACCG-3′ and 5′-AAGACAGATCTTCGGACTAGTGATCATTATGCGAGTGCGAC-3′ for Pf3D7_1200500 homology block 1; 5′-TATTTATTAAATCTAGAATTCGAGCAGTATTTTGGATCGTCC-3′ and 5′-ATTATTTTTACCGTTCCATGGCCCCAACATATACATTCACATAC-3′ for Pf3D7_1200500 homology block 2; 5′-AATACTCGCGGCCGCACTAGTCATGAACGCTTAAAGAAACAAGG-3′ and 5′-AAGACAGATCTTCGGACTAGTCCATTTCTATCTATACACTC-3′ for Pf3D7_1200600 homology block 1; 5′-TATTTATTAAATCTAGAATTCAGACCAATCGTTGAAAGCTG-3′ and 5′-ATTATTTTTACCGTTCCATGGGAGGGACATAATCTAATTTGG-3′ for Pf3D7_1200600 homology block 2; 5′-AATACTCGCGGCCGCACTAGTCATTCTGAAAATTGTTTGTATATGG-3′ and 5′-AAGACAGATCTTCGGACTAGTCACCCTAAACCTGTAAACCATAC-3′ for Pf3D7_0617400 homology block 1; and 5′-TATTTATTAAATCTAGAATTCGGAAGACATTGGAGAAAGTG-3′ and 5′-ATTATTTTTACCGTTCCATGGGCCCACCAATCTTCTCTTAG-3′ for Pf3D7_0617400 homology block 2.

### Isolation and analysis of parasite genomic DNA

DNA was isolated from 100 ml of culture at 5%–8% parasitemia. DNA was isolated using phenol–chloroform extraction followed by ethanol precipitation [51]. Specific recombination events were identified using PCR and the following primer pairs: for the amplifications shown in Fig 1, all reactions used the *hrp* specific primer 5′-GAACATAAAGTACAACATTAATATATAGC-3′ paired with 5′-GCCCATTCCTCGAACCA-3′ for integration at *var* gene Pf3D7_1200400; 5′- GAAGCATTTATAGAAGTAATGGAAC-3′ for integration at *rifin* gene Pf3D7_1200500; 5′- CCTCTATCCATTCTGTTAACC-3′ for integration at *var* gene Pf3D7_1200600; and 5′- GTAGGAGGATCTCCATCAAC-3′ for integration at *var* gene Pf3D7_0617400.

PCR conditions used throughout the study were initial denaturation at 95°C 5 min, followed by 32 cycles of 95°C for 15 seconds, 50°C for 15 seconds, and 60°C for 1 min. A final incubation at 65°C for 5 min terminated the reactions. The primer pairs shown in Figs 2 and 3 are P1forward 5′- GAGGGACATAATCTAATTTGG-3′ and P1reverse 5′- AGACCAATCGTTGAAAGCT-3′; P2forward 5′- CACCAAATATTCCTATGTGCAT-3′ and P2reverse 5′- CTCATTACGACAATAATAGATAGC-3′; P3forward 5′- CCTTGTTTCTTTAAGCGTTCATG-′ and P3reverse 5′- GGCAAATACAGGATTAGATATAAGG-3′; P4forward 5′- CCGCTCATATTTTCATGGTAC-3′ and P4reverse 5′- GTACCATGAAAATATGAGCGG-3′; P5forward 5′- TACTTCCTTCGCAGGGT-3′ and P5reverse 5′- GAGAAGAATAATAGGTCAAGAAAATG-3′; P6forward 5′- GGATATGATTGGAGAAGTTGTAC-3′ and P6reverse 5′- CCACCAATCTTCTCGTAATTG-3′; P7forward 5′- GGGAAACAATACGAGAACG-3′ and P7reverse 5′- GAATGTAGCAAAACCGATGCC-3′; and P8forward 5′- GTACGAAGATGAAACGCAC-3′ and P8reverse 5′- CCAACGTAATCGTTGAGG-3′.

### Sequencing of parasite genomic DNA and detection of recombination events

Parasite genomic DNA from clones D2, B10, and H10 was extracted using phenol–chloroform extraction, and sequencing libraries were prepared from 1 μg of unsheared gDNA (as measured using the Qubit dsDNA BR assay [Life Technologies, Carlsbad, CA, USA]) using the Oxford Nanopore Technologies ligation sequencing kit version 1D LSK 108 (Oxford, UK). This approach minimizes potential shearing of DNA template fragments and entirely avoids PCR artifacts by ligation of a manufacturer-supplied adapter nucleoprotein complex to native gDNA to facilitate loading of long library molecules into the protein nanopores. Nanopore sequencing was conducted with an Oxford Nanopore Technologies GridION X5 instrument using MIN-106/R9.4.1 flowcells. All samples were sequenced using a standard 48 hr run controlled by Oxford Nanopore Technologies MinKnow software, and raw data were basecalled with Albacore version 2.2.4. FASTQ files were extracted from basecalled FAST5 files with Poretools [52]. Alignments against the reference 3D7 genome sequence (v9.0) were performed using NGLMR [53] and visualized with IGV (version 2.4.16) for manual inspection. The left arm of chromosome 12 served as the initial site of observation, and recombination break points or telomere healing events were identified using the NCBI Blastn tool. All recombination and telomere healing events identified by BLAST were validated by PCR, followed by next-generation sequencing.

Parasite genomic DNA from clones VAR2CAL, RIF-FLIP-DCJ, and 700CAL-WT was extracted from parasites using phenol–chloroform extraction prior to library preparation. 5 μg of gDNA per library was processed using the PacBio 20-kb HMW gDNA ligation-based SMRTbell template prep kit (Pacific Biosciences, Menlo Park, CA, USA) with BluePippin (Sage Science, Beverly, MA, USA) size selection. Genomic DNA was sheared and ligated to sequencing adapters. Libraries were sequenced using v3SMRT Cells, and P6-C5 chemistry using an RSII instrument. SMRT Analysis software (Pacific Biosciences) was used to extract filtered fastq files from raw bax.h5 files produced by the RSII instrument using the subread filtering module in SMRT Analysis. Recombination break points and telomere healing events were validated using the strategy described above.

## Abbreviations

acs7: acyl-CoA synthetase 7
cam: calmodulin
CRISPR/Cas9: Clustered Regularly Interspaced Short Palindromic Repeats/CRISPR-associated protein-9 nuclease
dhfr: dihydrofolate reductase
DSB: double-strand break
FIKK: phenylalanine-isoleucine-lysine-lysine-motif–containing kinase
HR: homologous recombination
hrp: histidine-rich protein 2
NHEJ: nonhomologous end joining
PCR: polymerase chain reaction
PfEMP1: *P*. *falciparum* erythrocyte protein 1
Pfmc-2TM: *P*. *falciparum* Maurer’s cleft-2 transmembrane domain protein gene
RBC: red blood cell
rifin: repetitive interspersed family gene
stevor: subtelomeric variant open reading frame gene
TARE: telomere-associated repeat element

## References

[1] World Health Organization World Malaria Report 2018 [cited 2018 Dec 15]. Geneva, Switzerland: World Health Organization; 2018.

[2] Chen DS, Barry AE, Leliwa-Sytek A, Smith TA, Peterson I, Brown SM et al A molecular epidemiological study of var gene diversity to characterize the reservoir of Plasmodium falciparum in humans in Africa. PLoS ONE. 2011;6: e16629 10.1371/journal.pone.0016629 21347415 PMC3036650

[3] Otto TD, Bohme U, Sanders M, Reid A, Bruske EI, Duffy CW et al Long read assemblies of geographically dispersed Plasmodium falciparum isolates reveal highly structured subtelomeres. Wellcome Open Res. 2018;3: 52 10.12688/wellcomeopenres.14571.1 29862326 PMC5964635

[4] Barry AE, Leliwa-Sytek A, Tavul L, Imrie H, Migot-Nabias F, Brown SM et al Population Genomics of the Immune Evasion (var) Genes of Plasmodium falciparum. PLoS Pathog. 2007;3: e34 10.1371/journal.ppat.0030034 17367208 PMC1828697

[5] Lavstsen T, Salanti A, Jensen ATR, Arnot DE, Theander TG. Sub-grouping of Plasmodium falciparum 3D7 var genes based on sequence analysis of coding and non-coding regions. Malaria Journal 2003;2: 27 10.1186/1475-2875-2-27 14565852 PMC222925

[6] Kraemer SM, Smith JD. Evidence for the importance of genetic structuring to the structural and functional specialization of the Plasmodium falciparum var gene family. Molecular Microbiology. 2003;50: 1527–1538. 14651636 10.1046/j.1365-2958.2003.03814.x

[7] Smith JD, Chitnis CE, Craig AG, Roberts DJ, Hudson-Taylor DE, Peterson DS et al Switches in expression of *Plasmodium falciparum var* genes correlate with changes in antigenic and cytoadherent phenotypes of infected erythrocytes. Cell. 1995;82: 101–110. 7606775 10.1016/0092-8674(95)90056-x PMC3730239

[8] Baruch DI, Pasloske BL, Singh HB, Bi X, Ma XC, Feldman M et al Cloning the *P*. *falciparum* gene encoding PfEMP1, a malarial variant antigen and adherence receptor on the surface of parasitized human erythrocytes. Cell. 1995;82: 77–87. 7541722 10.1016/0092-8674(95)90054-3

[9] Su X, Heatwole VM, Wertheimer SP, Guinet F, Herrfeldt JV, Peterson DS et al A large and diverse gene family (*var*) encodes 200–350 kD proteins implicated in the antigenic variation and cytoadherence of *Plasmodium falciparum*-infected erythrocytes. Cell 1995;82: 89–100. 7606788 10.1016/0092-8674(95)90055-1

[10] Miller LH, Baruch DI, Marsh K, Doumbo OK. The pathogenic basis of malaria. Nature. 2002;415: 673–679. 10.1038/415673a 11832955

[11] Chattopadhyay R, Sharma A, Srivastava VK, Pati SS, Sharma SK, Das BS et al Plasmodium falciparum infection elicits both variant- specific and cross-reactive antibodies against variant surface antigens. Infection and Immunity. 2003;71: 597–604. 10.1128/IAI.71.2.597-604.2003 12540535 PMC145347

[12] Bull PC, Lowe BS, Kortok M, Molyneux CS, Newbold CI, Marsh K. Parasite antigens on the infected red cell surface are targets for naturally acquired immunity to malaria. Nat Med. 1998;4: 358–360. 9500614 10.1038/nm0398-358 PMC3836255

[13] Deitsch KW, Dzikowski R. Variant Gene Expression and Antigenic Variation by Malaria Parasites. Annu Rev Microbiol. 2017;71: 625–641. 10.1146/annurev-micro-090816-093841 28697665

[14] Ofori MF, Dodoo D, Staalsoe T, Kurtzhals JAL, Koram K, Theander TG et al Malaria-induced acquisition of antibodies to Plasmodium falciparum variant surface antigens. Infection and Immunity. 2002;70: 2982–2988. 10.1128/IAI.70.6.2982-2988.2002 12010988 PMC127986

[15] Tran TM, Li S, Doumbo S, Doumtabe D, Huang CY, Dia S et al An intensive longitudinal cohort study of Malian children and adults reveals no evidence of acquired immunity to Plasmodium falciparum infection. Clin Infect Dis. 2013;57: 40–47. 10.1093/cid/cit174 23487390 PMC3669526

[16] Bopp SE, Manary MJ, Bright AT, Johnston GL, Dharia NV, Luna FL et al Mitotic evolution of Plasmodium falciparum shows a stable core genome but recombination in antigen families. PLoS Genet. 2013;9: e1003293 10.1371/journal.pgen.1003293 23408914 PMC3567157

[17] Claessens A, Hamilton WL, Kekre M, Otto TD, Faizullabhoy A, Rayner JC et al Generation of antigenic diversity in Plasmodium falciparum by structured rearrangement of Var genes during mitosis. PLoS Genet. 2014;10: e1004812 10.1371/journal.pgen.1004812 25521112 PMC4270465

[18] Calhoun SF, Reed J, Alexander N, Mason CE, Deitsch KW, Kirkman LA. Chromosome End Repair and Genome Stability in Plasmodium falciparum. MBio. 2017;8: e00547–17. 10.1128/mBio.00547-17 28790200 PMC5550746

[19] Ghorbal M, Gorman M, MacPherson CR, Martins RM, Scherf A, Lopez-Rubio JJ. Genome editing in the human malaria parasite Plasmodium falciparum using the CRISPR-Cas9 system. Nat Biotechnol. 2014;32: 819–821. 10.1038/nbt.2925 24880488

[20] Wagner JC, Platt RJ, Goldfless SJ, Zhang F, Niles JC. Efficient CRISPR-Cas9-mediated genome editing in Plasmodium falciparum. Nat Methods. 2014;11: 915–918. 10.1038/nmeth.3063 25108687 PMC4199390

[21] Sander AF, Lavstsen T, Rask TS, Lisby M, Salanti A, Fordyce SL et al DNA secondary structures are associated with recombination in major Plasmodium falciparum variable surface antigen gene families. Nucleic Acids Res. 2014;42: 2270–2281. 10.1093/nar/gkt1174 24253306 PMC3936766

[22] Stanton A, Harris LM, Graham G, Merrick CJ. Recombination events among virulence genes in malaria parasites are associated with G-quadruplex-forming DNA motifs. BMC Genomics. 2016;17: 859 10.1186/s12864-016-3183-3 27809775 PMC5093961

[23] Claessens A, Harris LM, Stanojcic S, Chappell L, Stanton A, Kuk N et al RecQ helicases in the malaria parasite Plasmodium falciparum affect genome stability, gene expression patterns and DNA replication dynamics. PLoS Genet. 2018;14: e1007490 10.1371/journal.pgen.1007490 29965959 PMC6044543

[24] Huckaby AC, Granum CS, Carey MA, Szlachta K, Al-Barghouthi B, Wang YH et al Complex DNA structures trigger copy number variation across the Plasmodium falciparum genome. Nucleic Acids Res. 2019;47(4): 1615–1627. Epub 2018 Dec 21. 10.1093/nar/gky1268 30576466 PMC6393310

[25] Dzikowski R, Frank M, Deitsch K. Mutually Exclusive Expression of Virulence Genes by Malaria Parasites Is Regulated Independently of Antigen Production. PLoS Pathog. 2006;2: e22 10.1371/journal.ppat.0020022 16518466 PMC1386720

[26] Hamilton WL, Claessens A, Otto TD, Kekre M, Fairhurst RM, Rayner JC et al Extreme mutation bias and high AT content in Plasmodium falciparum. Nucleic Acids Res. 2017;45: 1889–1901. 10.1093/nar/gkw1259 27994033 PMC5389722

[27] Kirkman LA, Deitsch KW. Recombination and Diversification of the Variant Antigen Encoding Genes in the Malaria Parasite Plasmodium falciparum. Microbiol Spectr. 2014;2. 10.1128/microbiolspec.MDNA3-0022-2014 26104446

[28] Kirkman LA, Lawrence EA, Deitsch KW. Malaria parasites utilize both homologous recombination and alternative end joining pathways to maintain genome integrity. Nucleic Acids Res. 2014;42: 370–379. 10.1093/nar/gkt881 24089143 PMC3874194

[29] Fox BA, Ristuccia JG, Gigley JP, Bzik DJ. Efficient gene replacements in Toxoplasma gondii strains deficient for nonhomologous end joining. Eukaryot Cell. 2009;8: 520–529. 10.1128/EC.00357-08 19218423 PMC2669201

[30] Huynh MH, Carruthers VB. Tagging of endogenous genes in a Toxoplasma gondii strain lacking Ku80. Eukaryot Cell. 2009;8: 530–539. 10.1128/EC.00358-08 19218426 PMC2669203

[31] Scherf A, Mattei D. Cloning and characterization of chromosome breakpoints of Plasmodium falciparum: breakage and new telomere formation occurs frequently and randomly in subtelomeric genes. Nucleic Acids Res. 1992;20: 1491–1496. 10.1093/nar/20.7.1491 1579440 PMC312228

[32] Mattei D, Scherf A. Subtelomeric chromosome instability in Plasmodium falciparum: short telomere-like sequence motifs found frequently at healed chromosome breakpoints. Mutat Res. 1994;324: 115–120. 7517510 10.1016/0165-7992(94)90055-8

[33] Bottius E, Bakhsis N, Scherf A. Plasmodium falciparum telomerase: de novo telomere addition to telomeric and nontelomeric sequences and role in chromosome healing. Mol Cell Biol. 1998;18: 919–925. 10.1128/mcb.18.2.919 9447988 PMC108803

[34] Murillo SC, Akinyi OS, Abdallah JF, Pava Z, Dorado E, Incardona S et al Deletion of Plasmodium falciparum Histidine-Rich Protein 2 (pfhrp2) and Histidine-Rich Protein 3 (pfhrp3) Genes in Colombian Parasites. PLoS ONE. 2015;10: e0131576 10.1371/journal.pone.0131576 26151448 PMC4494814

[35] Kumar N, Pande V, Bhatt RM, Shah NK, Mishra N, Srivastava B et al Genetic deletion of HRP2 and HRP3 in Indian Plasmodium falciparum population and false negative malaria rapid diagnostic test. Acta Trop. 2013;125: 119–121. 10.1016/j.actatropica.2012.09.015 23041541

[36] Maltha J, Gamboa D, Bendezu J, Sanchez L, Cnops L, Gillet P et al Rapid diagnostic tests for malaria diagnosis in the Peruvian Amazon: impact of pfhrp2 gene deletions and cross-reactions. PLoS ONE. 2012;7: e43094 10.1371/journal.pone.0043094 22952633 PMC3429466

[37] Houze S, Hubert V, Le Pessec G, Le Bras J, Clain J. Combined deletions of pfhrp2 and pfhrp3 genes result in Plasmodium falciparum malaria false-negative rapid diagnostic test. J Clin Microbiol. 2011;49: 2694–2696. 10.1128/JCM.00281-11 21543573 PMC3147824

[38] Duffy MF, Byrne TJ, Carret C, Ivens A, Brown GV. Ectopic recombination of a malaria var gene during mitosis associated with an altered var switch rate. J Mol Biol. 2009;389: 453–469. 10.1016/j.jmb.2009.04.032 19389407 PMC3898907

[39] Sabatier L, Ricoul M, Pottier G, Murnane JP. The loss of a single telomere can result in instability of multiple chromosomes in a human tumor cell line. Mol Cancer Res. 2005;3: 139–150. 10.1158/1541-7786.MCR-04-0194 15798094

[40] Malkova A, Ira G. Break-induced replication: functions and molecular mechanism. Curr Opin Genet Dev. 2013;23: 271–279. 10.1016/j.gde.2013.05.007 23790415 PMC3915057

[41] Sakofsky CJ, Malkova A. Break induced replication in eukaryotes: mechanisms, functions, and consequences. Crit Rev Biochem Mol Biol. 2017;52: 395–413. 10.1080/10409238.2017.1314444 28427283 PMC6763318

[42] Li B. DNA double-strand breaks and telomeres play important roles in trypanosoma brucei antigenic variation. Eukaryot Cell. 2015;14: 196–205. 10.1128/EC.00207-14 25576484 PMC4346566

[43] Mason JMO, McEachern MJ. Mild Telomere Dysfunction as a Force for Altering the Adaptive Potential of Subtelomeric Genes. Genetics. 2018;208: 537–548. 10.1534/genetics.117.300607 29242289 PMC5788520

[44] Chen NWG, Thareau V, Ribeiro T, Magdelenat G, Ashfield T, Innes RW et al Common Bean Subtelomeres Are Hot Spots of Recombination and Favor Resistance Gene Evolution. Front Plant Sci. 2018;9: 1185 10.3389/fpls.2018.01185 30154814 PMC6102362

[45] Mason JMO, McEachern MJ. Chromosome ends as adaptive beginnings: the potential role of dysfunctional telomeres in subtelomeric evolvability. Curr Genet. 2018;64: 997–1000. 10.1007/s00294-018-0822-z 29589105

[46] Kraemer SM, Kyes SA, Aggarwal G, Springer AL, Nelson SO, Christodoulou Z et al Patterns of gene recombination shape var gene repertoires in Plasmodium falciparum: comparisons of geographically diverse isolates. BMC Genomics. 2007;8: 45 10.1186/1471-2164-8-45 17286864 PMC1805758

[47] Freitas-Junior LH, Bottius E, Pirrit LA, Deitsch KW, Scheidig C, Guinet F et al Frequent ectopic recombination of virulence factor genes in telomeric chromosome clusters of P. falciparum. Nature. 2000;407: 1018–1022. 10.1038/35039531 11069183

[48] Kirkman LA, Su XZ, Wellems TE. Plasmodium falciparum: isolation of large numbers of parasite clones from infected blood samples. Exp Parasitol. 1996;83: 147–149. 10.1006/expr.1996.0058 8654543

[49] Wu Y, Kirkman L, Wellems TE. Transformation of *Plasmodium falciparum* malaria parasites by homologous integration of plasmids that confer resistance to pyrimethamine. Proceedings of the National Academy of Sciences USA. 1996;93: 1130–1134. 10.1073/pnas.93.3.1130 PMC40043 8577727

[50] Deitsch KW, Driskill CL, Wellems TE. Transformation of malaria parasites by the spontaneous uptake and expression of DNA from human erythrocytes. Nucleic Acids Research. 2001;29: 850–853. 10.1093/nar/29.3.850 11160909 PMC30384

[51] Swamy L, Amulic B, Deitsch KW. Plasmodium falciparum var gene silencing is determined by cis DNA elements that form stable and heritable interactions. Eukaryot Cell. 2011;10: 530–539. 10.1128/EC.00329-10 21317310 PMC3127639

[52] Loman NJ, Quinlan AR. Poretools: a toolkit for analyzing nanopore sequence data. Bioinformatics. 2014;30: 3399–3401. 10.1093/bioinformatics/btu555 25143291 PMC4296151

[53] Sedlazeck FJ, Rescheneder P, Smolka M, Fang H, Nattestad M, von HA et al Accurate detection of complex structural variations using single-molecule sequencing. Nat Methods. 2018;15: 461–468. 10.1038/s41592-018-0001-7 29713083 PMC5990442

